# 3-(4-Meth­oxy­phen­yl)pyrido[2,3-*b*]pyrazine

**DOI:** 10.1107/S1600536810037943

**Published:** 2010-09-30

**Authors:** Cui-ping Wang, Jing-bo Yan, Hai-Jun Chi, Yan Dong, Zhi-qiang Zhang

**Affiliations:** a School of Chemical Engineering, University of Science and Technology Liaoning, Anshan, 114051, People’s Republic of China

## Abstract

In the title mol­ecule, C_14_H_11_N_3_O, the benzene ring is twisted by 14.0 (2)° from the plane through the fused ring system. In the crystal, π–π inter­actions [centroid–centroid distances = 3.609 (1), 3.639 (1) and 3.735 (1) Å] form stacks of mol­ecules propagating along the *b* axis. The crystal packing is further stabilized by weak inter­molecular C—H⋯O and C—H⋯N hydrogen bonds.

## Related literature

For a related structure, see: Koch *et al.* (2009 ▶). For the pharma­cological properties of quinoxaline compounds, see: Kleim *et al.* (1995 ▶); Abasolo *et al.* (1987 ▶); Rodrigo *et al.* (2002 ▶).
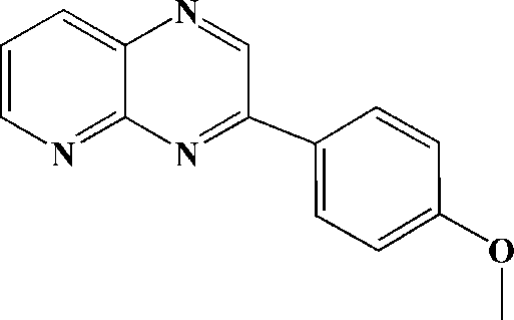


## Experimental

### 

#### Crystal data

C_14_H_11_N_3_O
*M* *_r_* = 237.26
Monoclinic, 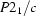

*a* = 6.4486 (13) Å
*b* = 7.3265 (15) Å
*c* = 24.216 (6) Å
β = 99.31 (3)°
*V* = 1129.0 (4) Å^3^
*Z* = 4
Mo *K*α radiation
μ = 0.09 mm^−1^
*T* = 153 K
0.20 × 0.18 × 0.12 mm

#### Data collection

Rigaku Saturn CCD area-detector diffractometer
Absorption correction: multi-scan (*CrystalClear*; Rigaku/MSC, 2005 ▶) *T*_min_ = 0.982, *T*_max_ = 0.989
9650 measured reflections
2677 independent reflections
2221 reflections with *I* > 2σ(*I*)
*R*_int_ = 0.034

#### Refinement

*R*[*F* ^2^ > 2σ(*F* ^2^)] = 0.049
*wR*(*F* ^2^) = 0.146
*S* = 1.09
2677 reflections
165 parameters
H-atom parameters constrained
Δρ_max_ = 0.39 e Å^−3^
Δρ_min_ = −0.27 e Å^−3^

### 

Data collection: *CrystalClear* (Rigaku/MSC, 2005 ▶); cell refinement: *CrystalClear*; data reduction: *CrystalClear*; program(s) used to solve structure: *SHELXS97* (Sheldrick, 2008 ▶); program(s) used to refine structure: *SHELXL97* (Sheldrick, 2008 ▶); molecular graphics: *SHELXTL* (Sheldrick, 2008 ▶); software used to prepare material for publication: *SHELXTL*.

## Supplementary Material

Crystal structure: contains datablocks global, I. DOI: 10.1107/S1600536810037943/cv2765sup1.cif

Structure factors: contains datablocks I. DOI: 10.1107/S1600536810037943/cv2765Isup2.hkl

Additional supplementary materials:  crystallographic information; 3D view; checkCIF report

## Figures and Tables

**Table 1:** Hydrogen-bond geometry (Å, °)

*D*—H⋯*A*	*D*—H	H⋯*A*	*D*⋯*A*	*D*—H⋯*A*
C6—H6⋯N3^i^	0.95	2.54	3.361 (2)	145
C3—H3⋯O1^ii^	0.95	2.44	3.123 (2)	129

Symmetry codes: (i) 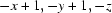; (ii) 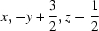.

**Fig. 1.**
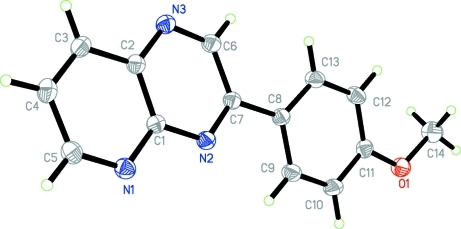
View of the molecule of (I) showing the atom-labelling scheme. Displacement ellipsoids are drawn at the 50% probability level.

## supplementary crystallographic information

### Comment

Functionalized quinoxalines represent an important class of nitrogen-containing heterocycle which display a broad spectrum of biological activity. Similar structure had been reported by Koch (Koch *et al.*, 2009). Quinoxaline derivatives were found to exhibit antimicrobial (Kleim *et al.* 1995), antitumor (Abasolo *et al.*,1987), and antituberculous activity (Rodrigo *et al.*, 2002). Here, we report the synthesis and crystal structure of the title compound, (I) (Fig. 1).

The molecular structure of title compound (I) is as shown in Fig.1. The dihedral angle between the pyrido[2,3-*b*]pyrazine ring and benzene ring is 14.0 (2)°. The O atom attached to the phenyl ring don't deviate the phenyl ring with an r.m.s deviation of 0.0047 (3) Å. As a result of π-π conjugation, the C*_sp_*^2^-O bond [O1—C9 = 1.3656 (13) Å] is significantly shorter than the C*_sp_*^3^-O bond [O1—C14 = 1.4266 (15) Å]. The crystal structure is stabilized by weak C—H···N and C—H···O intermolecular interactions and π-π interactions between the *Cg*1 (centroid of N1/C1—C5) and *Cg*2 (centroid of C1/C2/N3/C6/C7/N2). Selected geometric parameters are shown in Table 1.

### Experimental

A suspension of 2-(4-methoxyphenyl)-2-oxoacetaldehyde (2.0 mmol) and pyridine-2,3-diamine (3.0 mmol) in ethanol (5 ml) was stirred at room temperature. The reaction progress was monitored *via* TLC. The resulting precipitate was filtered off, washed with cold ethanol, dried and purified to give the target product as light yellow solid in 93% yield. Crystals of (I) suitable for single-crystal X-ray analysis were grown by slow evaporation of a solution in chloroform-ethanol (1:1).

### Refinement

All H atoms were positioned geometrically (C—H = 0.95–0.98 Å) and allowed to ride on their parent atoms, with *U*_iso_(H) = 1.2-1.5*U*_eq_ of the parent atom.

### Crystal data

**Table:**

C_14_H_11_N_3_O	*F*(000) = 496
*M**_r_* = 237.26	*D*_x_ = 1.396 Mg m^−^^3^
Monoclinic, *P*2_1_/*c*	Melting point: 428 K
Hall symbol: -P 2ybc	Mo *K*α radiation, λ = 0.71073 Å
*a* = 6.4486 (13) Å	Cell parameters from 3162 reflections
*b* = 7.3265 (15) Å	θ = 2.6–27.9°
*c* = 24.216 (6) Å	µ = 0.09 mm^−^^1^
β = 99.31 (3)°	*T* = 153 K
*V* = 1129.0 (4) Å^3^	Prism, colourless
*Z* = 4	0.20 × 0.18 × 0.12 mm

### Data collection

**Table:**

Rigaku Saturn CCD area-detector diffractometer	2677 independent reflections
Radiation source: rotating anode	2221 reflections with *I* > 2σ(*I*)
multilayer	*R*_int_ = 0.034
Detector resolution: 7.31 pixels mm^-1^	θ_max_ = 27.9°, θ_min_ = 1.7°
φ and ω scans	*h* = −8→8
Absorption correction: multi-scan (*CrystalClear*; Rigaku/MSC, 2005)	*k* = −9→9
*T*_min_ = 0.982, *T*_max_ = 0.989	*l* = −19→31
9650 measured reflections	

### Refinement

**Table:**

Refinement on *F*^2^	Secondary atom site location: difference Fourier map
Least-squares matrix: full	Hydrogen site location: inferred from neighbouring sites
*R*[*F*^2^ > 2σ(*F*^2^)] = 0.049	H-atom parameters constrained
*wR*(*F*^2^) = 0.146	*w* = 1/[σ^2^(*F*_o_^2^) + (0.0916*P*)^2^ + 0.0926*P*] where *P* = (*F*_o_^2^ + 2*F*_c_^2^)/3
*S* = 1.09	(Δ/σ)_max_ = 0.001
2677 reflections	Δρ_max_ = 0.39 e Å^−^^3^
165 parameters	Δρ_min_ = −0.27 e Å^−^^3^
0 restraints	Extinction correction: *SHELXL97* (Sheldrick, 2008), Fc^*^=kFc[1+0.001xFc^2^λ^3^/sin(2θ)]^-1/4^
Primary atom site location: structure-invariant direct methods	Extinction coefficient: 0.194 (16)

### Special details

**Table:**

Geometry. All e.s.d.'s (except the e.s.d. in the dihedral angle between two l.s. planes) are estimated using the full covariance matrix. The cell e.s.d.'s are taken into account individually in the estimation of e.s.d.'s in distances, angles and torsion angles; correlations between e.s.d.'s in cell parameters are only used when they are defined by crystal symmetry. An approximate (isotropic) treatment of cell e.s.d.'s is used for estimating e.s.d.'s involving l.s. planes.
Refinement. Refinement of *F*^2^ against ALL reflections. The weighted *R*-factor *wR* and goodness of fit *S* are based on *F*^2^, conventional *R*-factors *R* are based on *F*, with *F* set to zero for negative *F*^2^. The threshold expression of *F*^2^ > σ(*F*^2^) is used only for calculating *R*-factors(gt) *etc*. and is not relevant to the choice of reflections for refinement. *R*-factors based on *F*^2^ are statistically about twice as large as those based on *F*, and *R*- factors based on ALL data will be even larger.

### Fractional atomic coordinates and isotropic or equivalent isotropic displacement parameters (Å^2^)

**Table:**

	*x*	*y*	*z*	*U*_iso_*/*U*_eq_	
O1	0.71204 (14)	0.63090 (12)	0.31035 (3)	0.0269 (3)	
N1	1.25315 (15)	0.85321 (13)	0.01789 (4)	0.0217 (3)	
N2	1.03617 (15)	0.75236 (12)	0.07944 (4)	0.0194 (3)	
N3	0.73020 (15)	0.63878 (13)	−0.01163 (4)	0.0212 (3)	
C1	1.06896 (17)	0.77325 (15)	0.02545 (5)	0.0180 (3)	
C2	0.91672 (17)	0.71404 (15)	−0.02001 (5)	0.0187 (3)	
C3	0.95826 (18)	0.73760 (16)	−0.07480 (5)	0.0221 (3)	
H3	0.8603	0.6979	−0.1061	0.027*	
C4	1.14169 (19)	0.81838 (16)	−0.08199 (5)	0.0237 (3)	
H4	1.1734	0.8377	−0.1185	0.028*	
C5	1.28488 (19)	0.87344 (16)	−0.03427 (5)	0.0228 (3)	
H5	1.4127	0.9288	−0.0402	0.027*	
C6	0.70072 (18)	0.62433 (15)	0.04042 (5)	0.0211 (3)	
H6	0.5715	0.5753	0.0477	0.025*	
C7	0.85538 (17)	0.67952 (15)	0.08691 (5)	0.0181 (3)	
C8	0.81251 (17)	0.65886 (15)	0.14497 (5)	0.0193 (3)	
C9	0.93848 (19)	0.75321 (17)	0.18866 (5)	0.0245 (3)	
H9	1.0513	0.8266	0.1806	0.029*	
C10	0.90077 (19)	0.74085 (17)	0.24297 (5)	0.0253 (3)	
H10	0.9865	0.8063	0.2719	0.030*	
C11	0.73669 (19)	0.63212 (15)	0.25539 (5)	0.0210 (3)	
C12	0.61082 (19)	0.53616 (16)	0.21299 (5)	0.0235 (3)	
H12	0.4993	0.4616	0.2213	0.028*	
C13	0.65008 (18)	0.55069 (16)	0.15829 (5)	0.0227 (3)	
H13	0.5640	0.4852	0.1294	0.027*	
C14	0.5489 (2)	0.52103 (19)	0.32631 (5)	0.0320 (3)	
H14A	0.4121	0.5671	0.3081	0.048*	
H14B	0.5564	0.5260	0.3670	0.048*	
H14C	0.5662	0.3945	0.3147	0.048*	

### Atomic displacement parameters (Å^2^)

**Table:**

	*U*^11^	*U*^22^	*U*^33^	*U*^12^	*U*^13^	*U*^23^
O1	0.0318 (5)	0.0324 (5)	0.0180 (5)	−0.0071 (4)	0.0082 (3)	−0.0017 (3)
N1	0.0205 (5)	0.0225 (5)	0.0226 (5)	−0.0010 (4)	0.0050 (4)	−0.0004 (4)
N2	0.0200 (5)	0.0199 (5)	0.0185 (5)	−0.0010 (4)	0.0034 (4)	−0.0008 (4)
N3	0.0191 (5)	0.0233 (5)	0.0204 (5)	−0.0008 (4)	0.0013 (4)	0.0005 (4)
C1	0.0182 (5)	0.0172 (5)	0.0186 (6)	0.0012 (4)	0.0024 (4)	−0.0007 (4)
C2	0.0189 (5)	0.0167 (5)	0.0203 (6)	0.0017 (4)	0.0026 (4)	0.0005 (4)
C3	0.0245 (6)	0.0225 (6)	0.0185 (6)	0.0018 (4)	0.0008 (4)	0.0003 (4)
C4	0.0280 (6)	0.0241 (6)	0.0201 (6)	0.0021 (5)	0.0069 (4)	0.0019 (5)
C5	0.0218 (6)	0.0223 (6)	0.0253 (6)	−0.0002 (4)	0.0069 (4)	0.0002 (4)
C6	0.0183 (5)	0.0229 (6)	0.0220 (6)	−0.0020 (4)	0.0029 (4)	0.0001 (4)
C7	0.0185 (5)	0.0164 (5)	0.0194 (6)	0.0005 (4)	0.0032 (4)	−0.0010 (4)
C8	0.0203 (5)	0.0194 (5)	0.0183 (6)	−0.0003 (4)	0.0032 (4)	−0.0011 (4)
C9	0.0241 (6)	0.0278 (6)	0.0218 (6)	−0.0078 (5)	0.0046 (4)	−0.0012 (4)
C10	0.0277 (6)	0.0283 (7)	0.0192 (6)	−0.0066 (5)	0.0015 (5)	−0.0032 (5)
C11	0.0244 (6)	0.0216 (6)	0.0178 (6)	0.0007 (4)	0.0058 (4)	0.0000 (4)
C12	0.0242 (6)	0.0230 (6)	0.0247 (6)	−0.0053 (4)	0.0080 (5)	−0.0017 (5)
C13	0.0247 (6)	0.0229 (6)	0.0209 (6)	−0.0045 (4)	0.0048 (4)	−0.0041 (4)
C14	0.0340 (7)	0.0412 (8)	0.0241 (6)	−0.0085 (6)	0.0141 (5)	−0.0009 (5)

### Geometric parameters (Å, °)

**Table:**

O1—C11	1.3656 (13)	C6—H6	0.9500
O1—C14	1.4266 (15)	C7—C8	1.4838 (15)
N1—C5	1.3203 (15)	C8—C13	1.3924 (16)
N1—C1	1.3630 (14)	C8—C9	1.4069 (16)
N2—C7	1.3210 (14)	C9—C10	1.3786 (16)
N2—C1	1.3662 (14)	C9—H9	0.9500
N3—C6	1.3089 (15)	C10—C11	1.3955 (16)
N3—C2	1.3678 (15)	C10—H10	0.9500
C1—C2	1.4192 (16)	C11—C12	1.3921 (17)
C2—C3	1.4061 (15)	C12—C13	1.3922 (16)
C3—C4	1.3588 (17)	C12—H12	0.9500
C3—H3	0.9500	C13—H13	0.9500
C4—C5	1.4161 (17)	C14—H14A	0.9800
C4—H4	0.9500	C14—H14B	0.9800
C5—H5	0.9500	C14—H14C	0.9800
C6—C7	1.4361 (16)		
			
Cg1···Cg2^i^	3.639 (1)	Cg2···Cg2^ii^	3.735 (1)
Cg1···Cg2^ii^	3.609 (1)		
			
C11—O1—C14	118.35 (9)	C13—C8—C9	118.02 (11)
C5—N1—C1	116.75 (10)	C13—C8—C7	122.69 (10)
C7—N2—C1	116.94 (10)	C9—C8—C7	119.28 (10)
C6—N3—C2	116.34 (9)	C10—C9—C8	121.05 (11)
N1—C1—N2	116.73 (10)	C10—C9—H9	119.5
N1—C1—C2	122.39 (11)	C8—C9—H9	119.5
N2—C1—C2	120.88 (10)	C9—C10—C11	120.00 (11)
N3—C2—C3	119.72 (10)	C9—C10—H10	120.0
N3—C2—C1	121.51 (11)	C11—C10—H10	120.0
C3—C2—C1	118.75 (11)	O1—C11—C12	124.73 (11)
C4—C3—C2	118.49 (11)	O1—C11—C10	115.19 (10)
C4—C3—H3	120.8	C12—C11—C10	120.08 (11)
C2—C3—H3	120.8	C11—C12—C13	119.28 (11)
C3—C4—C5	119.06 (11)	C11—C12—H12	120.4
C3—C4—H4	120.5	C13—C12—H12	120.4
C5—C4—H4	120.5	C12—C13—C8	121.56 (11)
N1—C5—C4	124.56 (11)	C12—C13—H13	119.2
N1—C5—H5	117.7	C8—C13—H13	119.2
C4—C5—H5	117.7	O1—C14—H14A	109.5
N3—C6—C7	122.78 (11)	O1—C14—H14B	109.5
N3—C6—H6	118.6	H14A—C14—H14B	109.5
C7—C6—H6	118.6	O1—C14—H14C	109.5
N2—C7—C6	121.51 (11)	H14A—C14—H14C	109.5
N2—C7—C8	118.35 (10)	H14B—C14—H14C	109.5
C6—C7—C8	120.13 (10)		
			
C5—N1—C1—N2	−179.99 (9)	N3—C6—C7—N2	1.49 (17)
C5—N1—C1—C2	−0.10 (17)	N3—C6—C7—C8	−179.77 (10)
C7—N2—C1—N1	178.19 (9)	N2—C7—C8—C13	−166.08 (10)
C7—N2—C1—C2	−1.70 (16)	C6—C7—C8—C13	15.13 (16)
C6—N3—C2—C3	−178.71 (10)	N2—C7—C8—C9	14.63 (16)
C6—N3—C2—C1	−0.13 (17)	C6—C7—C8—C9	−164.15 (11)
N1—C1—C2—N3	−178.12 (9)	C13—C8—C9—C10	−0.79 (17)
N2—C1—C2—N3	1.77 (17)	C7—C8—C9—C10	178.54 (10)
N1—C1—C2—C3	0.47 (17)	C8—C9—C10—C11	0.59 (18)
N2—C1—C2—C3	−179.64 (9)	C14—O1—C11—C12	1.36 (17)
N3—C2—C3—C4	177.79 (10)	C14—O1—C11—C10	−179.27 (10)
C1—C2—C3—C4	−0.83 (17)	C9—C10—C11—O1	−179.45 (10)
C2—C3—C4—C5	0.83 (17)	C9—C10—C11—C12	−0.05 (18)
C1—N1—C5—C4	0.10 (17)	O1—C11—C12—C13	179.06 (10)
C3—C4—C5—N1	−0.48 (18)	C10—C11—C12—C13	−0.28 (17)
C2—N3—C6—C7	−1.43 (16)	C11—C12—C13—C8	0.06 (17)
C1—N2—C7—C6	0.18 (16)	C9—C8—C13—C12	0.46 (17)
C1—N2—C7—C8	−178.59 (9)	C7—C8—C13—C12	−178.84 (10)

Symmetry codes: (i) −*x*+2, −*y*+2, −*z*; (ii) −*x*+2, −*y*+1, −*z*.

### Hydrogen-bond geometry (Å, °)

**Table:**

*D*—H···*A*	*D*—H	H···*A*	*D*···*A*	*D*—H···*A*
C6—H6···N3^iii^	0.95	2.54	3.361 (2)	145
C3—H3···O1^iv^	0.95	2.44	3.123 (2)	129

Symmetry codes: (iii) −*x*+1, −*y*+1, −*z*; (iv) *x*, −*y*+3/2, *z*−1/2.
